# Intrapulmonary percussive ventilation via Mini-Trach II in critical care: a case report

**DOI:** 10.1186/s40981-021-00464-6

**Published:** 2021-08-06

**Authors:** Emina Niisato, Yoshiyuki Hiramoto, Hitoshi Yamada, Naoki Matsumiya

**Affiliations:** 1 Department of Anesthesiology and Intensive Care Medicine, Ibaraki Prefectural Central Hospital, 6528 Koibuchi, Kasama, Ibaraki 309-1703 Japan; 2 Department of Anesthesiology, Urayasu Hospital, Juntendo University, 2-1-1 Tomioka, Urayasu, Chiba 279-0021 Japan; 3 Department of Emergency and Intensive Care Medicine, Tsuchiura Kyodo General Hospital, 4‐1‐1 Otsuno, Tsuchiura, Ibaraki 300‐0028 Japan; 4 Department of Anesthesiology, Tsuchiura Kyodo General Hospital, 4‐1‐1, Otsuno, Tsuchiura, Ibaraki 300‐0028 Japan

**Keywords:** Intrapulmonary percussive ventilation, Mini-Trach II, Diaphragmatic injury, Diaphragmatic dysfunction, Atelectasis

## Abstract

**Background:**

Intrapulmonary percussive ventilation (IPV) facilitates the mobilization and clearance of bronchial secretions. Cricothyroidotomy using a Mini-Trach II device is a minimally invasive method used for secretion clearance. To our knowledge, there are no previous reports regarding IPV combined with Mini-Trach II.

**Case presentation:**

An 82-year-old man underwent controlled mechanical ventilation and IPV via an endotracheal tube to treat atelectasis following emergency surgical repair of a traumatic diaphragm laceration. He underwent cricothyroidotomy using Mini-Trach II for ensuring airway management after extubation. On resumption, IPV through a mouthpiece or face mask was unsuccessful owing to air leakage from his mouth. However, IPV via the already inserted Mini-Trach II could deliver the percussion flow and led to a marked improvement in his condition.

**Conclusion:**

This experience indicates that Mini-Trach II is beneficial as a minimally invasive interface for IPV that can deliver percussion flow efficiently.

## Introduction

Intrapulmonary percussive ventilation (IPV) is a modified method of intermittent positive-pressure breathing that superimposes high-frequency mini-bursts of air and aerosol on the patient’s intrinsic inspiratory and expiratory breathing pattern. This air burst creates an internal vibration (percussion) within the lungs and promotes the clearance of secretions [1]. IPV has been successfully used to improve gas exchange [2–5], atelectasis [6–8], and expiratory muscle performance [2]. Moreover, IPV has been reported to be effective in reducing the incidence of pneumonia [2], work of breathing [9], duration of mechanical ventilation [4], and length of intensive care unit (ICU) [4] and hospital stay [5, 10]. In a randomized controlled study [5] of patients with exacerbated chronic obstructive pulmonary disease, IPV was shown to circumvent the use of mechanical ventilation.

A mouthpiece, face mask, endotracheal tube, or tracheostomy can be used as a delivery interface for IPV. Mini-Trach II (Portex, Smiths Medical, Minneapolis, MN, USA) is a small-diameter tracheal tube used for minimally invasive cricothyroidotomy. Mini-Trach II is also useful to facilitate the management of airway secretions after extubation in patients who have undergone surgery or mechanical ventilatory support. To the best of our knowledge, there is no previous report of Mini-Trach II used as a delivery interface for IPV.

## Case presentation

**Fig. 1:**
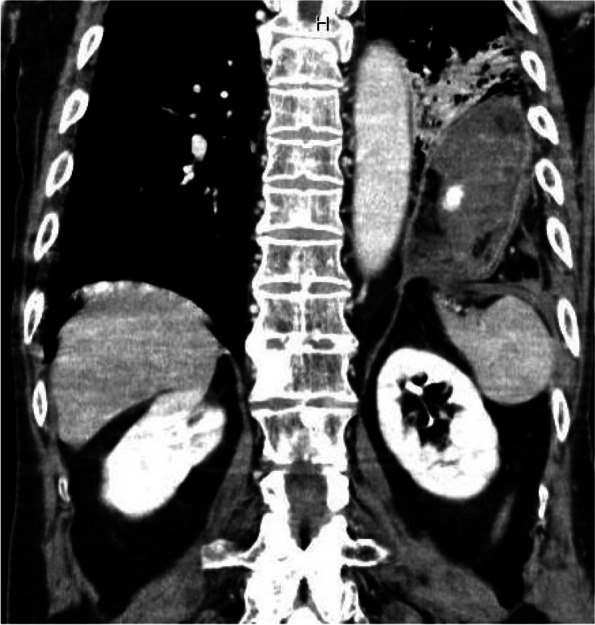
Preoperative chest computed tomography scans showing left diaphragmatic injury with hernia

An 82-year-old man (weight, 55 kg; height, 160 cm) in previously good health who was involved in a motor vehicle collision had left diaphragmatic injury with hernia (Fig. 1), and underwent urgent surgical diaphragm closure. The diaphragm had a laceration of approximately 15 cm, but no damage was detected in the abdominal organs or thoracic cavity other than the diaphragm. Oxygenation was maintained in controlled ventilation during surgery. Considering the possibility of respiratory failure after surgery, ventilation was controlled postoperatively in the ICU.

**Fig. 2:**
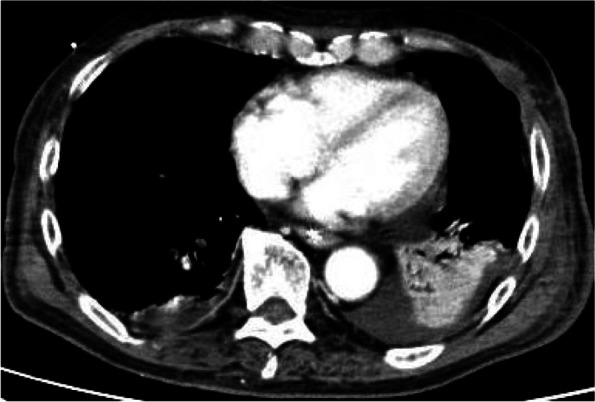
Chest computed tomography scans showing atelectasis in the left lower lung field on day 9, before intrapulmonary percussive ventilation therapy

Because of atelectasis in the left lower lung field and poor oxygenation, airway pressure release ventilation (APRV) was performed from day 3 and bronchoscopic aspiration was performed, but the atelectasis did not improve (Fig. 2); the patient had difficulty weaning from mechanical ventilatory support.

On day 15, IPV was delivered through the tracheal tube to treat persistent atelectasis of the left lower lung field due to left diaphragm elevation caused by diaphragmatic dysfunction. He received 15-min sessions of IPV daily in three different modes [easy (higher frequency), average, and hard (lower frequency)] for 5 min each with IPV (IPV-1c; Percussionaire Corp., Sandpoint, ID, USA). The nebulizer solution was composed of bromhexine hydrochloride and saline, and the working pressure was 30 psi.

**Fig. 3:**
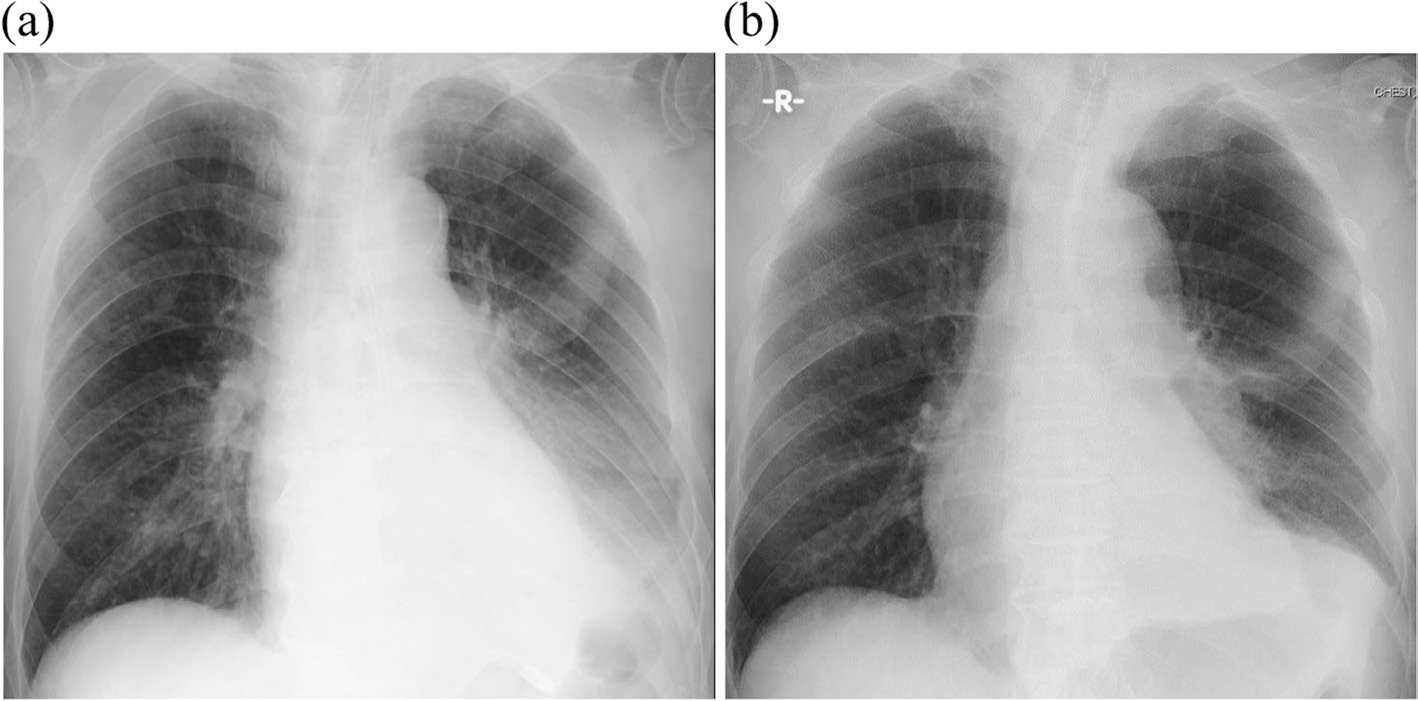
Chest radiograph showing reappearance of atelectasis in the left lower lung field on day 19, after extubation (**a**), and on day 30, after resolution following resumption of intrapulmonary percussive ventilation (**b**)

**Fig. 4:**
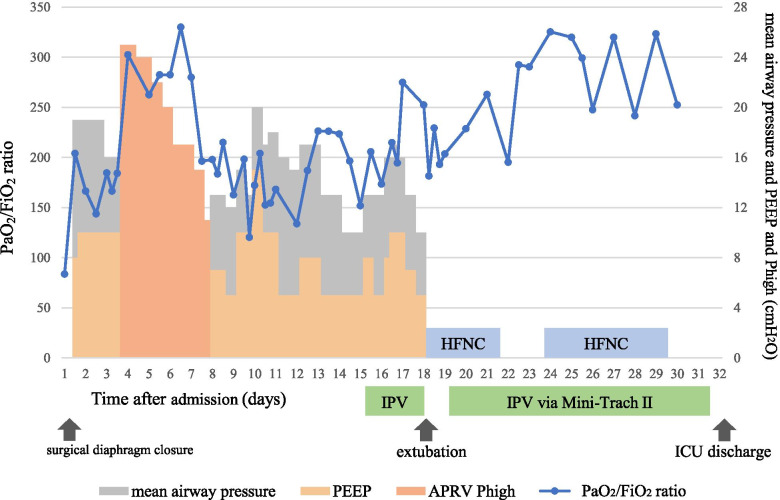
Clinical course of the patient after ICU admission. APRV, airway pressure release ventilation; HFNC, high-flow nasal cannula; ICU, intensive care unit; IPV, intrapulmonary percussive ventilation; PaO_2_/FiO_2_ ratio, the ratio of arterial oxygen partial pressure to fractional inspired oxygen; PEEP, positive end–expiratory pressure

After the start of IPV therapy, atelectasis of the left lower lung field improved. On day 18, atelectasis of the left lower lung field improved to the extent that he was extubated, but he was still coughing thick secretions. To facilitate endotracheal suctioning, he subsequently underwent cricothyroidotomy with Mini-Trach II. On the following day, chest radiograph revealed atelectasis of the same left lower lung field (Fig. 3a), and it was necessary to resume IPV to facilitate the clearance of secretions. Nevertheless, due to the large amount of air leakage from the corner of the mouth, a mouthpiece or face mask could not be successfully used as delivery interfaces for IPV. We decided to connect the IPV circuit to the already inserted Mini-Trach II because IPV delivered via an endotracheal tube or tracheostomy was thought to be invasive for him at that time. After connecting the IPV circuit to the Mini-Trach II, the amount of air leakage decreased, and auscultation and palpation confirmed that percussion flow had reached the peripheral lungs. Furthermore, the working pressure had increased to an effective level while performing IPV via Mini-Trach II. Accordingly, 15-min IPV sessions using Mini-Trach II were successfully conducted. Serial chest radiographs showed that the atelectasis of the left lower lung field resolved over the following 12 days (Fig. 3b), and he was discharged from the ICU on day 32. The clinical course of the patient is described in Fig. 4.

## Discussion

This report demonstrated that Mini-Trach II, although primarily designed for repeated endotracheal suctioning, can be used as a satisfactory and safe interface for IPV.

For both children and adults, IPV is used in the treatment of various diseases, including in patients with neuromuscular weakness who have difficulty eliminating sputum on their own [1, 10, 11]. The reduced capacity of sputum expectoration, as in our patients, is considered a good indication for IPV.

The interface for delivering IPV varies, and it is necessary to select the optimal device according to the patient’s condition. IPV is commonly performed through a face mask or a mouthpiece; however, a face mask is generally difficult to tolerate for patients, and in such cases, as the fitting of the mask to the face is insufficient, IPV cannot sufficiently increase the ventilation to an effective pressure. In the present case, because of air leakage from both the face mask and mouthpiece due to a defect of his teeth, we could not perform IPV effectively.

To the best of our knowledge, Mini-Trach II has not been employed previously to perform IPV. A randomized controlled trial of the addition of IPV therapy to the usual chest physiotherapy regimen in tracheostomized patients showed that IPV therapy improved gas exchange and expiratory muscle performance and reduced the incidence of pneumonia [2]. In addition, there have been some reports that IPV delivered through a tracheal tube with an inner diameter of approximately 4.0 mm improved atelectasis in pediatric patients [6, 12]. When IPV is connected to the Mini-Trach II with an inner diameter of 4.0 mm, the same effect can be expected as that when it is connected to a small-diameter tracheal tube.

Respiratory management using Mini-Trach II has the following advantages: insertion and removal operations can be performed easily and quickly, this approach is less invasive than tracheostomy, and it is easy to wean the patient from mechanical ventilatory support [13, 14]. Furthermore, as a result of early weaning from mechanical ventilatory support, rehabilitation can be actively promoted, and thus, the length of hospital stay can be shortened.

## Conclusions

We describe the case of a patient who showed improvement of persistent atelectasis after receiving IPV via Mini-Trach II. This case suggests that using Mini-Trach II for IPV can reduce the burden on patients, resulting in efficient delivery of percussion flow into the trachea and bronchi, thereby circumventing the need for tracheostomy in the case of continuing IPV after extubation.

## Data Availability

Not applicable.

## Abbreviations

APRV: Airway pressure release ventilation
HFNC: High-flow nasal cannula
ICU: Intensive care unit
IPV: Intrapulmonary percussive ventilation
PaO_2_/FiO_2_ ratio: The ratio of arterial oxygen partial pressure to fractional inspired oxygen
PEEP: Positive end–expiratory pressure
